# Coronavirus and paramyxovirus in bats from Northwest Italy

**DOI:** 10.1186/s12917-017-1307-x

**Published:** 2017-12-22

**Authors:** Francesca Rizzo, Kathryn M. Edenborough, Roberto Toffoli, Paola Culasso, Simona Zoppi, Alessandro Dondo, Serena Robetto, Sergio Rosati, Angelika Lander, Andreas Kurth, Riccardo Orusa, Luigi Bertolotti, Maria Lucia Mandola

**Affiliations:** 1 Istituto zooprofilattico sperimentale del Piemonte, Liguria e Valle d’Aosta, Via Bologna 148, 10148 Torino, Italy; 2 Robert Koch Institute, Seestraße 10, 13353 Berlin, Germany; 3 Chirosphera, via Tetti Barbiere 11, 10026 Santena, TO Italy; 4 Department of Veterinary Science, Largo Paolo Braccini 2, 10095 Grugliasco, TO Italy

**Keywords:** Bat-borne viruses, Coronavirus, Emerging viruses, Genetic characterization, Paramyxovirus, Surveillance

## Abstract

**Background:**

Bat-borne virus surveillance is necessary for determining inter-species transmission risks and is important due to the wide-range of bat species which may harbour potential pathogens. This study aimed to monitor coronaviruses (CoVs) and paramyxoviruses (PMVs) in bats roosting in northwest Italian regions. Our investigation was focused on CoVs and PMVs due to their proven ability to switch host and their zoonotic potential. Here we provide the phylogenetic characterization of the highly conserved polymerase gene fragments.

**Results:**

Family-wide PCR screenings were used to test 302 bats belonging to 19 different bat species. Thirty-eight animals from 12 locations were confirmed as PCR positive, with an overall detection rate of 12.6% [95% CI: 9.3–16.8]. CoV RNA was found in 36 bats belonging to eight species, while PMV RNA in three *Pipistrellus* spp. Phylogenetic characterization have been obtained for 15 alpha- CoVs, 5 beta-CoVs and three PMVs; moreover one *P. pipistrellus* resulted co-infected with both CoV and PMV. A divergent alpha-CoV clade from *Myotis nattereri SpA* is also described. The compact cluster of beta-CoVs from *R. ferrumequinum* roosts expands the current viral sequence database, specifically for this species in Europe. To our knowledge this is the first report of CoVs in *Plecotus auritus* and *M. oxygnathus*, and of PMVs in *P. kuhlii*.

**Conclusions:**

This study identified alpha and beta-CoVs in new bat species and in previously unsurveyed Italian regions. To our knowledge this represents the first and unique report of PMVs in Italy. The 23 new bat genetic sequences presented will expand the current molecular bat-borne virus databases. Considering the amount of novel bat-borne PMVs associated with the emergence of zoonotic infections in animals and humans in the last years, the definition of viral diversity within European bat species is needed. Performing surveillance studies within a specific geographic area can provide awareness of viral burden where bats roost in close proximity to spillover hosts, and form the basis for the appropriate control measures against potential threats for public health and optimal management of bats and their habitats.

## Background

Bats (order Chiroptera) represent at least one-fifth of existing mammals, consisting of over 1300 known species of which at least 44 are present in Europe [1] and 34 in Italy [2]. Species diversity is expected to increase as some taxa, i.e. *Myotis nattereri* complex, are in the processes of being defined as cryptic species using molecular approaches rather than using morphological characteristics [3]. Bats are grouped into two suborders: the fruit-eating megabats (Megachiroptera), or flying foxes consisting of the single family *Pteropodidae*, and the echolocating insectivorous microbats (Microchiroptera) comprising 16 bat families [4].

Bat borne viruses are arousing increased interest since viral infections in bats have been associated with zoonotic disease outbreaks in humans and domestic animals, including livestock. Rabies virus, Hendra and Nipah viruses, Severe Acute Respiratory Syndrome (SARS) and Middle East Respiratory Syndrome (MERS) coronaviruses, as well as Filoviruses exemplify the role of bats in spreading viruses [5–7].

In the last fifteen years, at least two widespread outbreaks have been caused by novel coronaviruses jumping the species barrier, SARS in 2002–2003 and MERS starting from the Arabian Peninsula since 2012 [6, 7]. Genetic similarities between the viral sequences detected during outbreaks and CoV sequences in bats suggest the viruses originated in flying mammals and presumably passed to humans through a previous adaptation in intermediate hosts, i.e. civet cats and dromedaries [8]. Coronaviruses (family *Coronaviridae*, subfamily *Coronavirinae*) are divided into four main genera: Alphacoronavirus (alpha-CoV) and Betacoronavirus (beta-CoV) found mainly in mammals, Gammacoronavirus detected in birds and marine mammals and Deltacoronavirus found mainly in birds. Several alpha and beta-CoVs have been described worldwide in different bat species (e.g. [9–17]). From the first report in China, *Rhinolophus* species have been specifically associated with SARS-like CoVs [18–20], belonging to the lineage b of beta-CoV genus. Further investigations are needed to clarify the origin of all mammalian coronaviruses, assumed to be from viral ancestors residing in bats [21], untill the recent discovery of a new and highly divergent CoV (i.e. WESV) from house shrews in China [22].

As of 2010, the circulation of CoV in Italian bat population has been notified in only few published studies: SARS-like beta-CoVs have been identified in *Rhinolophus* species [23] and CoVs sequences are available only for Italian *Pipistrellus kuhlii*, *Hypsugo savii*, *Nyctalus noctula*, *Epseticus serotinus*, *Myotis blythii* and *R. hipposideros* species from fecal samples [24, 25]. Despite the rapid accumulation of bat CoV sequences in the last decade, any viral isolation trial, on different mammalian and bat cell lines failed till 2013, when the first isolation of SARS-like CoV from bat fecal samples succeeded in China [26].

On the list of emerging zoonoses there is a broad diversity of bat-borne paramyxoviruses (PMV), belonging to the wide *Paramyxoviridae* family, as the emergent Nipah virus and Hendra virus (Henipaviruses) and rubulaviruses (e.g. Menangle virus, Tioman virus and Tuhoko virus 1, 2 and 3) (e.g. [27–29] and references therein). Detection and isolation of paramyxoviruses from tissues and urine have been obtained mainly from flying foxes of the genus Pteropus in Africa, Asia, and South America (e.g. [27, 30, 31]) and in Australia (e.g. [32–34]), but also microbat species not previously indicated as PMV reservoirs tested positive for PMV RNA in Africa and Europe [27, 35–37]. Moreover, the ever-increasing attention paid to bat-associated pathogens, has led to the discovery of numerous novel and yet unclassified PMV, revealing an unexpected genetic diversity in the *Paramyxovirinae* sub-family [36]. PMV identification has been reported in only few studies in insectivorous bats in Europe from Germany, Bulgaria, Romania and Luxembourg, with none of the novel viruses closely related with highly or human pathogenic paramyxoviruses [16, 17, 27, 36].

Following the increasing need of surveillance for bat-borne viruses and the wide range of bat species potentially representing reservoirs for known or unknown pathogens, this study aimed to estimate the viral diversity and distribution in the bat population resident in Northwest Italy. Our investigation was focused on coronaviruses and paramyxoviruses due to their proved ability to switch host and their zoonotic potential. Here we provide the phylogenetic characterization of viral polymerase gene fragments, which are highly conserved within the viral families under investigation.

## Methods

### Sites and sample collection

Since all bat species in Europe are protected under the Habitats Directive of the European Union [38] and the Agreement on the Conservation of Populations of European Bats [39], samples collection and bat species identification were performed by expert chiropterologists authorized by the Italian Ministry of Environment (authorization number DPN/2010/0011879 and 000882/PNM/08052014).

Bats were captured, during the three years of surveillance (2013–2016) in the Northwestern Italian regions of Piedmont and Liguria, following ethical and safety recommendations [40]. Samplings were conducted from mid-June to October, a period that approximately corresponds to the pregnancy, lactation, dispersion and mating activity of European bats. To minimize animal disturbance, bats were caught soon after parturition with nylon mist-nets of mesh size of 16 to 19 mm positioned at 10–20 m from the reproductive and temporary roost along flight paths towards foraging and drinking areas. During autumn catches were focused particularly at swarming sites in caves where individuals from different colonies meet to mate [41]. All nets were checked every 10 min and captured bats were removed carefully from nets as soon as possible to minimize injury, drowning, strangulation, or stress and individually placed into disposable cloth bags awaiting species identification, collection of biometric data and biological samples.

Species identification was carried out according to Dietz & Kiefer [1] and individual details such as age, class, sex, reproductive status, forearm length, and body mass were recorded. Saliva and urine drops, when present, were collected directly on the animal by swabbing, while feces were recovered, when present, from the cotton bag. All bats were released in the same place of capture after minimal manipulations and were not tagged.

Based on the results of the first two years of surveillance, an increase in feces collection was performed in 2016 setting up random, non-invasive feces samplings underneath single- species reproductive roosts. Briefly, plastic films were left on the ground under different areas of each reproductive colony, then 15 min later single fresh droppings were collected with clean disposable forks, placed in 1 ml of buffered peptone water and kept at 4 °C till analyses. Dead animals in good post-mortem conditions were also collected and stored at −20 °C for further analyses.

### RNA extraction and cDNA synthesis

Swabs and feces were maintained in 1 ml of UTM™ Viral Transport Medium (Catalog Number: 360C; Copan Diagnostics, Corona, California) and stored at −20 °C. Before any further analyses took place, the presence of the rabies virus antigen was investigated on dead animals by direct immunofluorescent staining in a BSL3 Laboratory, after necropsy. Once rabies infection has been excluded, samples underwent a pre-treatment before being submitted to automatic nucleic acid purification with magnetic beads.

Pre-treatment for tissues involved the preparation of a tissues pool composed by heart, lung, spleen and intestine from individual animals. The pools were homogenized at a ratio of 1:10 *w*/*V* in 1 ml of DEPC-treated PBS in a TissueLyser (Qiagen, Hilden, Germany). Tissue homogenates were then clarified at 13,000×g for 10 min at 4 °C, then 200 μl of tissues pool supernatant were incubated at 56 °C for 10 min with 180 μl of ATL buffer and 20 μl of Qiagen protease provided by the EZ1 Virus Mini Kit v2.0 (Qiagen, Hilden, Germany).To avoid any biosafety risk, the pre-treatment for swabs (saliva and urine) and feces suspensions involved the direct inactivation of 200 μl of each suspension in 200 μl of ATL buffer under a BSL3 hood. Nucleic acid purification (RNA/DNA) was finally accomplished on the EZ1 Advanced XL Instrument using an amount of 400 μl as sample input and a final elution volume of 60 μl of RNase-DNase free water, following the manufacturer’s guidelines. RNA was stored at −80 °C until amplification protocols were performed.

cDNA was synthetized from 5 μl of each RNA/DNA sample with the Transcriptor First Strand cDNA Synthesis Kit (Roche Diagnostics, Mannheim, Germany), according to manifacturer’s instructions.

### Coronavirus detection

For coronavirus detection, 2 μl of cDNA were amplified with an end-point PCR assay targeting a conserved RNA-dependent RNA polymerase (RdRp) gene fragment (537 bp), as described by Poon et al. [42]. The amplification was set up in a 25 μl reaction mixture containing 0.2 mM deoxynucleoside triphosphates, 1.5 mM MgCl2, 0.2 μM of IN-6 and IN-7 primer and 1 U of Platinum Taq Polymerase (Invitrogen, Carlsbad, CA). The cycling conditions were 94 °C for 2 min, 40 cycles at 94 °C for 1 min, 48 °C for 1 min, 72 °C for 1 min and final elongation step at 72 °C for 7 min.The annealing temperature of primer was modified from 58 °C to 48 °C.

Upon amplification, 20 μl of PCR products were run in 1.5% agarose gel electrophoresis and visualized by GelGreen Nucleic Acid Gel Stain (Biotium) staining; bands of the expected size were excised from the gel for sequencing.

### Paramyxovirus detection

For paramyxovirus detection, a broadly reactive seminested PCR assay specific for the RNA polymerase (L)- gene (538 bp) of the *Paramyxovirinae* subfamily was applied. 0 2 μl of cDNAs were amplified using the PAR primers designed by Tong et al. [43] and the protocol optimized with Taguchi method by Kurth et al. (36). Briefly for first round, the final concentration of the 25 μl reaction mix was: 0.1 mM deoxynucleoside triphosphates, 10 mM MgCl2, 0.12 μM of PAR F1 and PAR R primers and 1.25 U of Platinum Taq Polymerase (Invitrogen, Carlsbad, CA). The cycling conditions were 94 °C for 2 min, 40 cycles at 94 °C for 15 s, 50 °C for 30 s, 72 °C for 30 s and a final elongation step at 72 °C for 7 min. Then 1 μl first round PCR product was used in the second round with the same concentrations except for the MgCl_2,_ set up at 1 mM and the use of PAR F2 and PAR R primers, cycling parameters were identical to the first round.

PCR products (20 μl) were run and recovered from a 1.5% agarose gel, as described before.

### Sequencing and phylogenetic analysis

Amplicons were purified by gel extraction with the QIAquick Gel Extraction kit (Qiagen, Hilden, Germany), according to the manufacturer’s instructions. After elution, nucleic acid quantification of the recovered DNA was done using Thermo Scientific Nanodrop spectrofotometer and submitted for direct sequencing to BMR Genomics, Padua, Italy. The obtained chromatograms were manually checked for unclear base calls and edited using Geneious R7.1.7 software (Geneious, Auckland, New Zealand).

The sequences were aligned using Muscle (implemented in Geneious software) and the alignment was used to evaluate the best evolutionary model (Modeltest ver 3.7) and to draw a bayesian phylogenetic tree (MrBayes ver. 3.1.2). Consensus tree was created after at least 1 million of heuristic search generations and after eliminating the first 25% of evaluated tree topologies (burnin = 25%).

### Biomolecular species identification

A total genomic DNA extraction was performed only for PCR positive individuals starting from the original swab suspensions using the QIAmp DNA Mini kit (Qiagen, Hilden, Germany) and following the manufacturer protocol. To confirm species identification by genetic determinations, the complete mitochondrial Cytochrome b gene (Cytb) was amplified as in Puechmaille et al. [3]. PCR products were submitted for direct sequencing to BMR Genomics, Padua, Italy. The obtained chromatograms were manually checked for unclear base calls and edited using Geneious R7.1.7 software (Geneious, Auckland, New Zealand). Species identification was conducted by comparing the obtained sequences to on-line available reference sequences (BLAST alignment, NCBI web site).

## Results

### Samples collection

Starting from June 2013 till October 2016 a total of 302 animals (35 dead; 267 live) belonging to 19 bat species were collected during 49 capture sessions in 38 locations of Piedmont and five of Liguria regions. Collection of saliva, urine and feces from the same animal was not possible for each of the 267 live bats handled, leading to the final collection of 123 oral swabs (37%), 49 urine swabs (15%) and 158 fecal drops (48%). Sex definition was determined for 195 bats: 117 males and 78 females; the additional 107 single fecal droppings collected in 2016 under 4 different monospecific colonies were considered as non-assigned individual samples. All captured species are listed in Table 1.

**Table 1:** Sampled bat species and CoV and PMV prevalences detected

Genus	Species	n°sampled (n° pos)	CoV detection; n/N (%)	PMV detection; n/N (%)
*Pipistrellus*	*Pipistrellus kuhlii*	56 (4)	2/56; 3.6%	2/56; 3.6%
	*Pipistrellus pipistrellus*	20 (5)	4/20; 20%	1/20; 5%
	*Pipistrellus nathusii*	2		
*Myotis*	*Myotis myotis*	43 (4)	4/43; 9.3%	
	*Myotis brandtii*	1		
	*Myotis bechsteinii*	1		
	*Myotis nattereri*	22 (3)	3/22; 13,6%	
	*Myotis daubentonii*	24 (2)	2/24; 8.3%	
	*Myotis emarginatus*	29		
	*Myotis oxygnathus*	23 (2)	2/23; 8.7%	
	*Myotis mistacinus*	3		
*Hypsugo*	*Hypsugo savii*	5		
*Plecotus*	*Plecotus auritus*	14 (1)	1/14; 7.1%	
	*Plecotus austriacus*	1		
	*Plecotus macrobullaris*	1		
	*Barbastella barbastellus*	17		
*Nyctalus*	*Nyctalus leisleri*	1		
*Rhinolophus*	*Rhinolophus ferrumequinum*	38 (18)	18/38; 47.4%	
	*Rhinolophus hipposideros*	1		
	Total	302 (39)	36/302; 12% [95% CI: 9.6–17]	3/302; 1% [95% CI: 0.3–3,1]

95% Confidence Interval (95% CI) is expressed only for CoV and PMV overall rates

No animal captured during the active surveillance showed signs of disease. During necropsies, no macroscopic lesions referring to infectious diseases were observed, and all the examined bats were negative in the rabies virus antigen IF test.

### Coronavirus and paramyxovirus detection

CoV and PMV positive sample types included feces (33/158; 21%) and urine swabs (6/49; 12.2%). None of the tissue pools from dead bats or oral swabs were PCR positive. A significantly greater percentage of female bats, 11.5% (9/78), were PCR-positive than males, 4.3% (5/117), (*p* = 0.05).

**Fig. 1:**
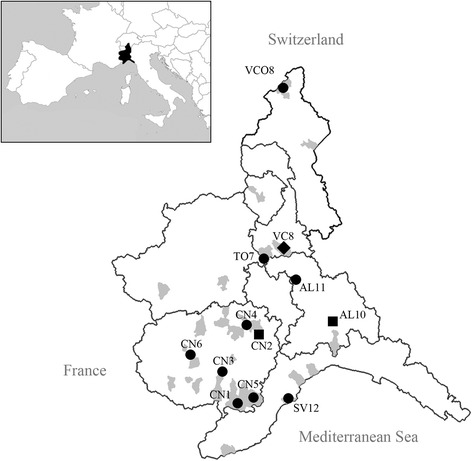
Map of Piedmont and Liguria sites where a CoV or PMV sequence was detected. Circles represent CoV positive sites; squares identify PMV positive sites and diamonds represent the site positive for both CoV and PMV. Sites are identified according to a code formed by the province abbreviation and progressive numbers, i.e. in Piedmont, for Cuneo province CN1: Ormea, CN2: Rodello, CN3: Pianfei, CN4: Santa Vittoria d’Alba, CN5: Garessio CN6: Villar San Costanzo; for Torino province TO7: Verrua Savoia; for Vercelli province VC8: Trino; for Verbano-Cusio-Ossola province VCO9: Baceno; for Alessandria province AL10: Tassarolo, AL11: Vignale Monferrato; in Liguria, for Savona province SV12: Finale Ligure. Sampled municipalities that were found negative are reported in grey.

Coronavirus and/or paramyxovirus RNA was found in 38 animals belonging to eight bat species (Table 1). Specifically, CoV RNA was detected in 36 bats from 12 sampling sites in Piedmont and one in Liguria, while PMV RNA in three animals from three sampling sites in Piedmont; a map showing the positive sites is presented in Fig. 1. In our sample set, the detection rate of CoV was 12% (36/302; 95% confidence interval [CI] = 9.6–17) ranging between 3.6% for *P. kuhlii,* despite representing the most abundant species in our sample, and 47.4% for *R. ferrumequinum*.

Phylogenetic analysis was performed on 20 unique sequences obtained from 36 samples that yielded a PCR product of the expected size after the CoV PCR screening. The positive samples were collected from: *M. nattereri* (*n* = 3), *M. myotis* (*n* = 2), *M. oxygnathus* (*n* = 1), *P. kuhlii* (*n* = 1), *P. pipistrellus* (*n* = 3), *P. auritus* (*n* = 1) and *R. ferrumequinum* (*n* = 9). Any new sequences identified were submitted to GenBank and the accession numbers assigned are given in Table 2. The PMV strains were detected in three different provinces from two *P. kuhlii* at CN2 and AL10 sites and one *P. pipistrellus* at VC8 site; moreover, phylogenetic analysis based on the L-gene fragment was possible for all the three strains retrieved in this study. Interestingly, one *P. pipistrellus* from VC8 site was coinfected by both CoV and PMV as PCR positive results were obtained from the same urine sample. Details of positive sequenced samples are displayed in Table 2.

**Table 2:** CoV and PMV positive samples for which a sequence is available

Species	ID	Sample type	Capture date	Site	Setting^a^	Sex/age^b^	CoV sequence (AN)/CoV genus	PMV sequence (AN)
*Myotis nattereri*	560	Feces	31/08/13	CN1	T roost	M/ad	Mnat560_IT_13 (KY780381)/alpha	
	562	Urine				F/juv	Mnat562_IT_13 (KY780382)/alpha	
	1021	Feces	16/08/14	TO7	R roost	F/juv	Mnat1021_IT_14 (KY780387)/alpha	
*Pipistrellus pipistrellus*	1015	Urine	05/08/14	VC8	R roost	F/ad	Ppip1015C_IT_14 (KY780385)/alpha	Ppip1015P_IT_14 (KY780403)
	1016	Feces				F/juv	Ppip1016_IT_14 (KY780386)/alpha	
	1000	Feces	11/08/14	VC9	Fora-ging	M/ad	Ppip1000_IT_14 (KY780384)/alpha	
*Pipistrellus kuhlii*	600	Feces	19/08/14	CN2	R roost	F/ad		Pkuh600_IT_14 (KY780401)
	605	Feces				F/ad	Pkuh605_IT_14 (KY780383)/alpha	
	621	Urine	06/08/14	AL10	R roost	F/ad		Pkuh621_IT_14 (KY780402)
*Myotis myotis*	4658	Feces	15/08/16	CN4	R roost		Mmyo4658_IT_16 (KY780397)/alpha	
	4663	Feces					Mmyo4663_IT_16 (KY780398)/alpha	
*Myotis oxygnathus*	4235	Feces	06/07/16	SV12	R roost		Moxy4235_IT_16 (KY780395)/alpha	
*Plecotus auritus*	4241	Feces	20/09/16	CN5	Swar-ming	M/ad	Paur4241_IT_16 (KY780396)/beta	
*Rhinolophus ferrumequinum*	4009	Feces	04/07/16	CN6	R roost		Rfer4009_IT_16 (KY780388)/alpha	
	4011	Feces					Rfer4011_IT_16 (KY780389)/alpha	
	4015	Feces					Rfer4015_IT_16 (KY780390)/alpha	
	4019	Feces					Rfer4019_IT_16 (KY780391)/beta	
	4024	Feces					Rfer4024_IT_16 (KY780392)/alpha	
	4025	Feces					Rfer4025_IT_16 (KY780393)/alpha	
	4027	Feces					Rfer4027_IT_16 (KY780394)/beta	
	4674	Feces	13/07/16	AL11	R roost		Rfer4674_IT_16 (KY780399)/beta	
	4675	Feces					Rfer4675_IT_16 (KY780400)/beta	

ID: Identification number corresponds to the progressive and unique number assigned to each analyzed sample. Site codes are displayed in Fig. 1

^a^the setting where bats were caught, R roost: reproductive roost; T roost: temporary roost

^b^age definitions are juv: juvenile and ad: adult

### CoV phylogeny

**Fig. 2:**
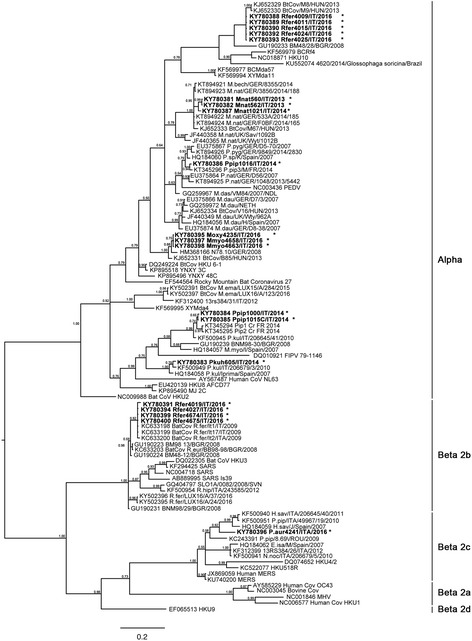
Bayesian phylogenetic tree of alpha and beta-CoVs derived from bats and other species. Representative RdRp sequences were extracted from GenBank and the alignment carried out on 372 nucleotides for a total of 101 sequences: twenty original and 81 available from Genbank, among the alpha and beta-CoVs genera. Members of betacoronaviruses are separated into four lineages, 2a, 2b, 2c and 2d. Posterior probability values of the clades are reported above branches. The CoVs name in the tree is composed by the sequence GenBank accession number plus the name of the strain. Our 20 new sequences are reported in bold and labeled with a star (*)

RdRp phylogeny is presented in Fig. 2 and shows that 15 CoV strains from this study clustered in the alphacoronavirus genus and 5 in the beta-coronavirus genus.

As shown in Fig. 2, the three *M. nattereri* alpha-CoV strains (560, 562 site CN1 and 1021 site TO7) cluster with nucleotide similarities ranging from 94 to 96% within a CoV clade composed of three *M. nattereri* and one *M. bechstenii* from Germany (AN: KT94921–924) and another *M. nattereri* from Hungary (AN: KJ652333), but show only an 86% identity with *M. nattereri* CoVs strains from UK 2009.

Genetic species determination based on the Cyt B gene fragment of 837 bp for these *M. nattereri* species showed a 99% sequence identity with a French *M. nattereri* isolate (AN: JF412408) named “MspA Mnat22 cytochrome b gene” was highlighted.

Based on this finding, our new CoVs strains belong to the *M. nattereri SpA*, a putative new species within the *M. nattereri* species complex.

Three alpha-CoV strains found in feces samples of three bats belonging to the *Myotis* genus show 100% identity to each other (4235 from *M. oxygnathus*, site SV12 and 4658 and 4663 from *M. myotis*, site CN4) and form a divergent clade. When compared to other CoVs, this clade showed the highest identity (~97%) with two *M. myotis* CoV strains, from Germany and Hungary (AN: HM368166 and KJ652331).

Two *P. pipistrellus* CoV sequences (1000 site VCO9 and 1015 site VC8) cluster together with two *P. pipistrellus* strains (Pip1, Pip2) from the same species detected in France in 2014 (AN: KT345294–95) and one *P. pipistrellus* strain from Italy (AN: KF500945); interestingly the third *P. pipistrellus* CoV (1016 site VC8) is ~27% divergent from the others and clusters near Pip3 CoV strain from France (AN: KT345296).

The *P. kuhlii* CoV sequence clusters (605 site CN2) with a similarity of ~97%, within a clade of two *P. kuhlii* strains from Italy 2007 (AN: KF500949) and Spain (AN: HQ184058).

Five *R. ferrumequinum* alpha-CoV sequences (4009, 4011, 4015, 4024, 4025 site CN6) found in fecal droppings from the same monospecific roost, showed 100% identity with each other clustering within the clade formed by the only three *R. ferrumequinum* alpha-CoV sequences detected in Europe so far, 3% divergent from the ones from Hungary (AN: KJ652329–30) and 13% from the Bulgarian one (AN:GU190233).

Among the beta-CoV group (lineage b) four *R. ferrumequinum* CoV strains (4019, 4027 site CN6 and 4674, 4675 site AL11) cluster together with other three Italian beta-CoV sequences from the same species (AN: KC33198–200). Interestingly, the 4027 sequence is 100% identical with 4674 and 4675, although originating from two *R. ferrumequinum* roosts located at 130 km distance.

One novel beta-CoV sequence from *Plecotus auritus* (AN: KY780396) clusters separately in the beta-CoV group (lineage c) showing only a ~88% similarity with two *H. savii* CoV strains one from Spain (AN: HQ184059) and one from Italy (AN: KF500940) and a *P. pipistrellus* strain from Italy (AN: KF500951). It’s divergence from a MERS CoV strain isolated in 2014 from a camel (AN: KU740200) is 14%. Phylogenetic analyses of this short fragment show that CoVs cluster based on the relatedness of host species.

### PMV phylogeny

**Fig. 3:**
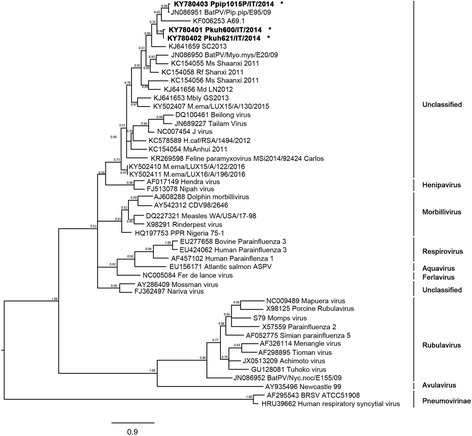
Bayesian phylogenetic tree of the *Paramixoviridae* family. The tree is built on a L-gene fragment of 393 nucleotides on a total of 48 taxa: three original sequences and 45 sequences. Available L-gene sequences, representative of the seven currently known and unclassified genera of the *Paramixovirinae* sub-family, together with two strains from the *Pneumovirinae* sub-familiy were extracted from GenBank. Posterior probability values of the clades are reported above branches. The samples name in the tree is composed by the GenBank accession number plus the name of the strain. New obtained sequences are sequences are reported in bold and labeled with a star (*)

PMV phylogeny based on representative L-gene sequences available from GenBank is presented in Fig. 3.

The new PMV strains were detected in three different locations from one *P. pipistrellus* at VC8 site and two *P. kuhlii* bats at CN2 and AL10 sites (80 km distance).

Our 1015 *P. pipistrellus* strain revealed a 97% nucleotide identity with the E95 PMV strain (AN: JN086951) detected in the same bat species in Germany in 2009, but is more than 23% divergent from any other known PMV sequence.

The two PMV sequences from *P. kuhlii* (600 site CN2 and 621 site AL10) are 97% similar to each other and cluster separately from previously known PMV sequences (18–20% divergence) in the L-gene fragment phylogenetic tree.

To our knowledge, the *P. kuhlii* species was never previously implicated as paramyxovirus host.

## Discussion

Recently, emerging disease surveillance programs have intensified to investigate the role of bats in the evolution and spillover of zoonotic pathogens from wildlife. Our study involved three years of active and passive surveillance to characterize the viral diversity of the Northwestern Italian bat population. Using viral family-wide PCRs we identified and phylogenetically characterized 20 new CoVs and 3 PMVs strains. To date, studies on bat CoVs phylogeny are mainly based on datasets of short sequences (i.e. 440 bp) (e.g. [9, 10, 13, 14, 24, 25, 44]) due to the difficulties of obtaining isolates and good quality viral RNA from bats, but ideally long sequence fragments would be beneficial to infer more reliable phylogenies.

The high prevalence of positive fecal samples (21%) in our study is in concordance with other studies, which identified feces as the best sample type for CoVs detection in bats [9, 18]. Rather than collecting samples from individually caught bats, which is time consuming and labor intensive, collecting single fecal droppings under mono-species roosts turned out to be a reliable and non-invasive method for virological surveillance of bat roosts during their reproductive period. Moreover, urine is confirmed as the most suitable and appropriate sample types for detection of paramyxoviruses in bat populations [33], considering that 2 out of the 3 PMV positive samples from our study were urine swabs.

In 2013–2014 coronavirus circulation was identified in at least four species-specific reproductive roosts of Piedmont: TO7 site for *M. nattereri*, VC8 site for *P. pipistrellus*, CN2 and AL10 for *P. kuhlii*. Unfortunately, attempts to re-test the same roosts in 2016 failed since the VC8 colony moved due to the effect of human disturbance (i.e. robbery of the copper roof cover used as refuge by *P. pipistrellus* bats), and the other three colonies located in private buildings were inaccessible due to logistical reasons. The likelihood of roost disturbance should be taken into account when putting in place bat surveillance plans to enable a steady follow up of the colonies over time.

Bats social behavior could explain the significantly higher infection rate detected in our study for female bats all sampled in August near maternity roosts. Previous studies documented higher virus detection rates in females and juveniles captured near maternity roosts in summer, supporting the hypothesis that virus amplification occurs mainly in reproductive roosts [11, 45].

The identification of the same CoV strains (100% identical) in different roosts of the same bat species (i.e. *R. ferrumequinum* and *P. pipistrellus*) located also at over 100 km distance, seems to confirm that most bat-CoVs appear species-specific and thus more closely associate with the host species than the sampling location [11, 15, 20]. Interestingly, we identified a divergent alpha-CoV lineage in *M. nattereri SpA*, representing a cryptic lineage within the *Myotis nattereri* species complex in the Mediterranean region. The lineage is known to be present in Italy, however no information is available for Germany and Hungary [46]. Following the host-virus coevolution theory based on their close phylogenetic concordance [47], the small divergence (from 3.5 to 5%) between our *M. nattereri SpA* CoV strains and the German or Hungarian *M. nattereri* ones could indicate that they all reside in the *M. nattereri SpA* host, considering that molecular species identification for those specimens is lacking.

The detection of identical alpha-CoV sequences in two different species belonging to the *Myotis* genus (*M. oxygnathus* and *M. myotis*) from two distinct roosts (sites SV12 and CN4) 90 km apart could be due to the expansion and overlapping of habitats and foraging areas of *Myotis* spp. through the Maritime Alpine chain and valleys. To our knowledge this is the first report of CoV in the *M. oxygnathus* species.

The compact cluster of almost identical beta-CoV (lineage b) strains from two separate *R. ferrumequinum* roosts gives further indications that the *Rhinolophus* genus may represent the specific host for SARS-like CoVs and gives an important contribution in terms of available beta-CoV sequences from this species in Europe. To our knowledge, this is the first report of CoV in the *P. auritus* species. This sequence clusters separately within the beta-CoV (lineage c) showing a 14% divergence with a MERS strain identified from a camel in Egypt.

The detection of highly divergent alpha-CoV strains within one *P. pipistrellus* reproductive roost, the circulation of both alpha and beta-CoVs within one *R. ferrumequinum* roost and the co-infection of *P. pipistrellus* with both CoV and PMV provide further evidence that bats are able to carry more than one virus. While infection with multiple CoVs in the same species/bat/colony is well known, and has been previously reported in China [26, 48, 49] and Europe [19], apart from metagenomic studies notably biased towards the identification of sequences from dsDNA viruses, to our knowledge the coinfection of different ssRNA viral families in the same animal was so far reported only in one study in Europe from *P. pygmaeus* in Hungary [44]. In the specific, the coinfection with two ssRNA viral families within the same host may be explicable in the light of the IFN inhibition used by paramyxoviruses to circumvent host’ innate immune response [50]. This mechanism, known as IFN antagonism, may be exploited by other viruses able to escape the adaptive immunity, e.g. CoVs, to be introduced and proliferate in the same host, as observed in mallards [51].

By the increased viral surveillance, a considerable number of novel paramyxoviruses has been discovered in pteropoid and non-pteropoid species, but to date the number of bat PMV sequences for Europe is very scarce and only from few bat species [16, 17, 27, 36]. The three new PMV strains, two in *P. kuhlii* and one in *P. pipistrellus* species, couldn’t be classified within any of the current seven known PMV genera, but cluster in the crowded, unassigned PMV clade, which comprises several bat derived strains. Our report represents the first identification of PMVs in the *P. kuhlii* species worldwide. The two sequences, retrieved from two roosts located 90 km apart, are divergent from previously known PMV clusters, which may indicate a stronger association to the host species rather than the geographic area also for paramyxoviruses. This viral tropism is also strongly supported by the high similarity of our *P. pipistrellus* sequence to that of the one other E95 PMV sequence retrieved in Germany from the same bat species. In support of this hypothesis, a study on renal tissues from African bats underlined how paramyxovirus divergence in pteroid and non-pteroid bats correlates with bat taxonomy, suggesting a strong association with bat genera [37]. Because the L-gene fragment used as genetic marker in the aforementioned study is not overlapping with the sequence we used, we couldn’t phylogenetically compare them. Nevertheless, given the high similarity our *P. pipistrellus* sequence shows with the E95 PMV strain, our findings support this association. Moreover, an extensive collection of urine samples from the colony would be necessary to facilitate PMVs isolation, which remains a critical requirement for full genome and pathogenic characterization of the strains detected.

## Conclusions

Compared to previous studies published in Italy [24, 25], we detected alpha and beta-CoVs in not previously surveyed Italian regions and in new bat species; moreover, this report represents the first and novel identification of PMVs in Italy. The 23 new bat genetic sequences will fill gaps and expand the current molecular bat-borne virus databases.

Considering the amount of novel bat-borne PMVs associated with the emergence of zoonotic infections in animals and humans in the last years define the virus diversity within European bat species is needed. Performing surveillance studies within a specific geographic area can provide awareness of viral burden where bat roosts are in close proximity to spillover hosts, and can form the basis for the appropriate control measures to curb potential threats for public health and optimal management of bats and their habitats.
